# Impact of a population based intervention to increase the adoption of multiple physical activity practices in centre based childcare services: a quasi experimental, effectiveness study

**DOI:** 10.1186/1479-5868-9-101

**Published:** 2012-08-29

**Authors:** Meghan Finch, Luke Wolfenden, Maryann Falkiner, Danielle Edenden, Nicole Pond, Louise L Hardy, Andrew J Milat, John Wiggers

**Affiliations:** 1 Population Health, Hunter New England Health District, Booth Building, Longworth Avenue, Wallsend, 2287, Australia; 2 School of Medicine and Public Health, University Drive, Callaghan, 2308, Australia; 3 Physical Activity, Nutrition and Obesity Research Group, Medical Foundation Building, The University of Sydney, Sydney, 2006, Australia; 4 Evidence and Evaluation Branch, NSW Ministry of Health, Miller St, North Sydney, 2060, Australia; 5 School of Public Health, Medical Foundation Building, The University of Sydney, Sydney, 2006, Australia

**Keywords:** Daycare centers, Preschool, Childcare, Children, Physical activity, Policy, Practice

## Abstract

**Background:**

There is considerable scope to improve the delivery of practices that increase the physical activity of children in centre based childcare services. Few studies have reported the effectiveness of interventions to address this, particularly at a population level. The primary aim of this study was to describe the impact of an intervention to increase the adoption of multiple policies and practices to promote physical activity in centre based childcare services.

**Methods:**

A quasi experimental study was conducted in centre based childcare services (n =228) in New South Wales (NSW), Australia and involved a three month intervention to increase the adoption of eight practices within childcare services that have been suggested to promote child physical activity. Intervention strategies to support the adoption of practices included staff training, resources, incentives, follow-up support and performance monitoring and feedback. Randomly selected childcare services in the remainder of NSW acted as a comparison group (n = 164) and did not receive the intervention but may have been exposed to a concurrent NSW government healthy eating and physical activity initiative. Self reported information on physical activity policies, fundamental movement skills sessions, structured physical activity opportunities, staff involvement in active play and provision of verbal prompts to encourage physical activity, small screen recreation opportunities, sedentary time, and staff trained in physical activity were collected by telephone survey with childcare service managers at baseline and 18 months later.

**Results:**

Compared with the comparison area, the study found significantly greater increases in the prevalence of intervention services with a written physical activity policy, with policy referring to placing limits on small screen recreation, and with staff trained in physical activity. In addition, non-significant trends towards a greater increase in the proportion of intervention services conducting daily fundamental movement skill sessions, and such services having a physical activity policy supporting physical activity training for staff were also evident.

**Conclusion:**

The intervention was effective in improving a number of centre based childcare service policies and practices associated with promoting child physical activity. Adoption of a broader range of practices may require more intensive and prolonged intervention support.

## Background

Adequate physical activity among young children promotes bone health, is protective against obesity and is beneficial for child social, psychological and fundamental motor skill development [1-4]. Despite these benefits, international research suggests that many children aged less than five years do not meet current recommendations for participation in physical activity, exhibit high levels of sedentary behavior, and participate in excessive television viewing [5-8].

Centre based childcare services, such as preschools and long day care services [9] represent a promising setting for the delivery of interventions to increase the physical activity levels of children [10,11] as they provide access to a large number of preschool age children (three to five years old), often for prolonged periods [9,12]. In Australia, for example, centre based childcare is provided by both long day care and preschool services with 95% of children attending either a full-day preschool or long day care services in the year before commencing formal schooling [9,13]. Furthermore, such childcare services have existing organizational infrastructure and equipment that can be used to promote physical activity [11,14] and are supported by accreditation and licensing guidelines that require services to promote the health and physical development of children [15,16].

Findings from descriptive research conducted in centre based childcare services have identified a range of characteristics associated with increased child physical activity. Specifically, children attending services with higher quality facilities and equipment [17-19], lower playground density (less children per square meter) [20], with more vegetation, unbroken open areas [18] and with staff trained in physical activity [17,19,21,22] have been found to be more active. Similarly, children are more likely to be active if they attend centre based childcare services with a physical activity policy [19,22]; that deliver structured physical activities [10,17,19,23]; that support fundamental movement skill development [2,24]; where small screen recreation opportunities are limited [14,25]; where staff are involved in, and verbally prompt children’s active play [26,27]; and where there is adequate availability of portable play equipment [19]. While experimental research is limited, findings from centre based physical activity interventions suggests that multi-component interventions which seek to address a number of these practices are effective in increasing child physical activity whilst in care [23]. As such, the implementation of physical activity promoting policies and practices are recommended by best practice guidelines for the sector [28].

Despite the potential to increase child physical activity, previous studies indicate that centre based childcare services do not comply with the recommended physical activity promoting practices [29-31]. A recent Australian study, for example, found that only half of preschool and long day care services had a physical activity policy (41-48%); 28-30% of services allowed children to view non active small screen recreation daily; and 49-51% did not have any staff who had recently participated in physical activity training [29]. Similarly, in the U.S, it has been reported that just 25% of staff in centre based childcare services had completed training in physical activity, 86% of services provided less than two hours of active play time each day and 61% of childcare service staff did not participate in active play with children [30].

A recent review of obesity interventions in centre based childcare [32] has identified just three trials of interventions primarily targeting the adoption of obesity prevention policies and practices [33-35]. Of these trials, two evaluated interventions targeting the adoption of nutrition practices only [34,35]. The remaining study, a randomized controlled trial of 84 services, assessed the effectiveness of an intervention to promote the adoption of both physical activity and nutrition policies and practices [33].

The intervention in this study was the U.S Nutrition and Physical Activity Self-Assessment for Child Care program, and consisted of a service environmental self-assessment tool, education workshops and the provision of technical support for staff. The program failed to significantly improve self-reported adoption of physical activity policies and practices [33,36]. A further relevant peer reviewed trial identified by the authors randomly allocated 15 preschools to receive an intervention comprising of a staff professional development workshop, service resources and access to a health promotion officer to support healthy eating and physical activity practice adoption [37]. Following the intervention, the service manager self- reported frequency of fundamental movement skill sessions significantly increased relative to control services, yet there were no between group differences on five other measures of the physical activity environment [37].

Given the limited number of published population-based interventions in this setting [38], we conducted a study to describe the impact of an intervention to increase the adoption of multiple physical activity promoting policies and practices in childcare services. What distinguished this study from previous research is the scale of the intervention and its assessment of setting wide adoption of these practices. We also sought to determine the impact of the intervention on childcare service manager’s knowledge of physical activity recommendations and the acceptability of the intervention strategies to managers.

## Methods

### Study design and setting

A quasi experimental study was conducted in centre based childcare centers in the state of New South Wales (NSW), Australia. All centre based childcare services in one region (Hunter New England) were offered the intervention. Randomly selected childcare services in the remainder of the state acted as a comparison and were exposed to a separate government physical activity intervention. The intervention region involved a large non-metropolitan area (more than 130 000 km^2^) encompassing urban and rural communities (based on the Australian Standard Geographic Classification system) [39] with a population of 60,970 children aged 0–5 years (12% of NSW 0–5 year old population and 23% of the state’s Indigenous children aged 0–4) [40].

The comparison region of NSW has an area of 801 305 km2 and includes major cities, inner regional centers, outer regional centers, remote and very remote areas. NSW has a population of 506 095 children aged 0–5 years (33% of the Australian children’s population and 31% of the country’s Indigenous children) [41].The study was approved by the Hunter New England Human Research Ethics Committee (HNEHREC 06/07/26/4.04).

### Sample and recruitment

The sampling frame consisted of all centre based childcare services in the state as recorded by the licensing agency for such services. In this study centre based childcare services were defined as long day care services and preschools. In Australia, long day care services provide centre based care for eight or more hours per day for five days per week and usually enroll children aged from six weeks old up to six years. Preschools provide centre based care for six to eight hours per day and enroll children aged between three to six years. Both long day care services and preschools provide educational activities for children aged 3–5 years to assist in their preparation for school. Across Australia the role and function of preschools and long day care services are similar [9] and licensing and accreditation requirements regarding physical activity policies and practices identical [42]. Furthermore research suggests that the current prevalence of implementation of physical activity promoting policies and practices for both services are alike [29]. Those services catering solely for children with special needs such as intellectual or physical disabilities were excluded from the study (n = 28).

All eligible centre based childcare services (n = 338) located within the intervention region were invited to participate in the intervention. A ten percent simple random sample of eligible centre based childcare services in the remainder of the state were invited to participate in the study to serve as a comparison group (n = 268). Managers of all eligible services were sent a letter inviting them to participate in the study. Approximately two weeks after receipt of the letter, a trained research assistant telephoned each service to assess their interest in participation and confirm their eligibility.

### Intervention

**Figure 1:**
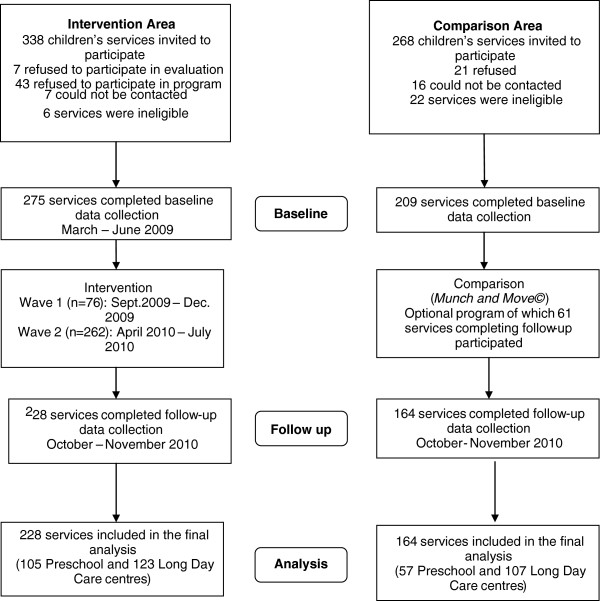
Participant recruitment and retention by group.

The intervention was designed by the authors (MF, LW, DE, NP and MF)in conjunction with a regional community advisory group with representation from local service managers, health promotion practitioners, early childhood researchers and physical activity experts. The timing of intervention delivery was also determined by the research team and was conducted as a component of a large scale regional child obesity prevention initiative ( http://www.goodforkids.nsw.gov.au) offered to all centre based childcare services within a defined geographic government health district. The same intervention was delivered over a three month period to services across the intervention region in two waves. Approximately 40% of services received the intervention between September and December 2009 (wave one). The remaining services received the intervention between April and July 2010 (wave two). The timeline for delivery of the intervention can be seen in Figure 1.

Eight practices that have been reported to promote child physical activity [43] and that were consistent with the Australian National Physical Activity Best practice guidelines for Early Childhood Services [28] were targeted by the intervention for adoption by the services for children 3–5 years. Multiple implementation strategies, selected based on theory and evidence of efficacy, were offered to childcare services to facilitate their adoption of the physical activity promoting policies and practices described above. Specifically, the five strategies employed were:

1. Offer of staff training [22,44]: Services were invited to send two staff to a six hour physical activity training workshop. The choice of staff to attend was at the discretion of each service and could include the service manager or teachers or a combination of the two. Staff training was conducted by a respected early childhood training organization, and a local service manager and academic with considerable expertise in child physical activity. The training provided basic information, skill development and guidance regarding service physical activity policies and practices and how they could be modified to better support child activity in care. All services were provided access to an online web- based training module covering similar content to that provided in the workshop. Service managers were encouraged to ensure all service staff who had not attended the workshop completed the online module. The online module required approximately 40 minutes of staff time.

2. Offer of information, program resources and instructional materials [45,46]: Program resources and instructional materials were delivered in the form of a resource package. This included, a guide manual with background and instructional information covering topics related to key physical activity promoting practices, three age appropriate structured activities handbooks, two DVDs demonstrating fundamental movement skills, laminated game cards and staff lanyards with pictorial and descriptive explanations of fundamental movement skills, a planning poster which identified timeframes for services to implement practice changes and, a fundamental movement skills template to assist with programming fundamental movement skills sessions. All printed resources are available to download from the *Good for Kids. Good for Life.* program website http://www.goodforkids.nsw.gov.au.

3. Offer of follow-up support [47,48]: Service managers were offered two 15 minute telephone support calls to reinforce key program messages, identify barriers to practice change and provide additional advice and support. Calls were delivered after staff had attended training or the service received an intervention resource kit via post. Services also received two support emails or faxes and six newsletters to reinforce key messages, case study successful services and provide further information to services based on barriers identified through telephone contacts. Twenty percent of services elected to provide a fax number, rather than email as their contact. All services were provided with a free contact number direct to a member of the project team for any further queries or support.

4. Provision of performance monitoring and feedback regarding practice adoption [47,49]: Information collected during the telephone support contacts with the service was used to monitor adoption of intervention components and provide performance feedback regarding individual service implementation during telephone contacts.

5. Offer of incentives [50,51]. Services adopting a physical activity policy went in a draw to win vouchers for educational toys and resources and services with staff completing on-line training also went in a draw to win vouchers for educational toys and resources. Staff completing online training went in a draw to win holiday accommodation.

### Comparison group

Centre based childcare services in the comparison area had the opportunity to participate in an alternative, government delivered intervention (*Munch and Move ©*http://www.healthykids.nsw.gov.au/campaigns…/about-munch-move.aspx) that aimed to promote physical activity and healthy eating in childcare services. The intervention was offered to all comparison area centre based childcare services in two waves, with preschools being offered the program from June 2008 and long day care services from August 2010 [52,53]. The strategies employed to support adoption of physical activity nutrition practice changes involved service staff being invited to attend a full day workshop provided by a non-government organization, provision of a printed resource folder and provision of a small financial grant to support staff attendance at training or the purchase of equipment. The opportunity existed for additional support strategies to be provided by local health promotion services at their discretion.

### Data collection procedures

A 30 minute computer-assisted telephone interview (CATI) was developed by the research team to determine the study outcomes and assess intervention acceptability. The instrument was developed with advice from an advisory group consisting of centre based childcare service managers, NSW Department of Community Services, NSW Ministry of Health, health promotion practitioners, pediatric researchers and physical activity experts.

Service managers in intervention and comparison area centre based childcare services participated in the CATI. Baseline assessments were conducted from March to June 2009 and follow-up assessments occurred from September to October 2010. Follow-up was conducted approximately12 months after the initiation of the intervention with wave one services and approximately six months after the initiation of the intervention for wave two services. In Australia service managers are responsible for policy development, ensuring compliance with licensing and accreditation requirements. Furthermore most service managers also have teaching roles, and as such would have knowledge of practices.

## Measures

### Service characteristics

Service size (average number of children enrolled), operational characteristics (average opening hours per day, number of days per week open), number of university trained teachers, number of primary contact staff (teaching staff or educators, not including cooks, administration staff) and, number of Aboriginal and Torres Strait Islander child enrollments for services in the intervention and comparison areas were reported by the service managers. Service postcode was used to describe the socioeconomic and geographic remoteness of the service location [54,55]. A remoteness index was used to describe the geographic locality of services. The index classifies post codes based on physical access to a range of goods and services and opportunities for social interaction. Major cities are classified as highly accessible, inner regional areas have some restrictions to accessibility; outer regional areas have significantly restricted accessibility and remote areas have very restricted accessibility [56].

### Physical activity practices

Survey items assessing physical activity practices can be seen in Table 1. The items were developed following a review of existing validated U.S tools [57,58] and were designed to match the specific practices targeted by the intervention. All survey items were reviewed for suitability and pre-tested by centre based childcare service managers. The survey items have been previously used to report on service physical activity policies and practices in Australia [29].

**Table 1:** Physical activity policy and practice survey items and measures

**Telephone survey item**	**Response option**	**Formation of measure**	**Measure descriptor and supporting references**
Does your service have a written policy on physical activity?	Yes; No; Don’t know	% of services that responded yes	1. Services with a physical activity policy [19,22]
Does your policy specifically refer to development of fundamental movement skills?	Yes; No; Don’t know	% of services that responded yes	a) Physical activity policy referring to child fundamental movement skills development
Does your policy specifically refer to limits on small screen recreation & TV?	Yes; No; Don’t know	% of services that responded yes	b) Physical activity policy referring to limits on small screen recreation and TV
Does your policy specifically refer to staff training in physical activity?	Yes; No; Don’t know	% of services that responded yes	c) Physical activity policy referring to physical activity training for staff
Does your service carry out planned, adult guided sessions to facilitate preschool age children’s exploration and development of fundamental movement skills? This would include structured teacher led activity during which children explore and practice one or more Fundamental Movement Skills	Yes; No	% of services that:	2. Services conducting daily fundamental movement sessions with recommended components [28,43]
		· responded yes to carrying out sessions;	
		and	
		· responded that sessions were conducted once per day	
		and	
		· responded that sessions always included; warm up, cool down, skill specific feedback, extension and challenge experiences, modeling and demonstration.	


How often do the fundamental movement skills sessions occur?	Once per day; 4 times per; 3 times per week ; 2 times per week; Once per week ; Less than once per week ; Don't know		
How often do fundamental movement skills sessions include each of the following components? Warm up & cool down activities? Skill specific feedback e.g. error detection and correction? Extension and challenge experiences? Staff modeling and demonstration?	Always; Very often; Sometimes; Rarely; Never		





How much of your daily operating time is spent in a form of specific adult guided activity such as group music, dancing or planned fundamental movement skills sessions with preschool age children?	Hours and minutes recorded	Mean hours	3. Time spent on structured physical activities [10,17,19,23]


On a usual day do primary contact staff join in and participate with preschool age children during child initiated free active play? This is when staff join in with active play that the children initiated and are leading and would include activities such as a staff member pushing a child on a swing while talking to another staff member. Please note general supervision while standing still is not considered role modelling.	Yes; No; Don’t know	% of services that:	4. Services where all staff usually participate in free active play (role modeling) [26,27]
		· responded yes to primary contact staff joining in and participating with children during child initiated free active play;	
		and	
		· responded that all staff implement this practice	


How many primary contact staff implement this practice?	All staff; Most staff ; Some staff		
On a usual day do primary contact staff provide verbal prompts to encourage or extend preschool age children’s activity during child initiated free active play by saying things like 'run hard', 'good throw', or 'can you do it again'?	Yes; No; Don’t know	% of services that:	5. Services where all staff usually provide verbal prompts for physical activity [26,27]
		· responded yes to primary contact staff providing verbal prompts to encourage or extend children’s activity during child initiated	
		and	
		· responded that all staff implement this practice	

How many primary contact staff implement this practice?	All staff; Most staff ; Some staff		
On average, how often are preschool age children allowed to watch small screen (e.g. television, videos or DVDs or have time to play computer games) where they are sitting still?	Once per day; 4 times per week ; 3 times per week ; 2 times per week; Once per week; Less than once per week; Never	% of services that answer yes to less than once per week	6. Services where children are allowed to watch Small screen recreation less than once per week [14,25]
This question is about occasions during the day where the MAJORITY of children are sitting still for more than 30 minutes at a time, for example times where staff put toys on a table and children are only allowed to sit at the table and play, or group activities where children are seated on the floor. On average, excluding meal and nap times, how many occasions during the day would this occur?	Never; Once per day; 2 times per day; 3 times per day; 4 times per day; 5 times per day; Don't know	% of services that responded never	7. Services where children participate in seated activities for no longer than 30 minutes at a time [14]


Next I would like to ask you some questions about any professional development relating to physical activity attended by your staff. development relating to physical activity attended by your staff. In the last 12 months have any staff at your service participated in professional development or specific training relating to physical activity provided by an agency external to your service?	Yes; No; Don’t know	% of services that responded yes	8. Services with staff trained in physical Activity [19,21]

### Service manager knowledge of physical activity recommendations

Service managers were asked to report the recommended minutes/hours for: minimum time for participation in physical activity per day for children aged two to five years; the maximum time for participation in small screen recreation for children aged two to five years; and, the maximum time for children aged two to five being sedentary per day (based on the Australian National Physical Activity Recommendations for Children aged 0–5 years) [59].

### Acceptability of the intervention strategies and resources

The managers in the intervention area were asked to respond to a series of statements assessing the acceptability of the program on a five-point Likert scale (strongly agree, agree, disagree, strongly disagree and neutral). These statements included whether staff perceived that children at their service benefited from their involvement in the physical activity intervention; whether they would recommend the intervention to other services, and whether the training workshop was beneficial for staff to attend.

Acceptability of the support calls was assessed by asking managers to respond on a four-point Likert scale (very useful, somewhat useful, not at all useful) to the statement: ‘Overall, how useful did you find the support calls were in helping your service to implement best practice physical activity strategies at your service?’. The acceptability of each of the intervention resources (game cards, lanyards, activity handbooks, DVDs, guide manual and policy template) was similarly assessed (very useful, somewhat useful, not at all useful).

### Analyses

All analyses were conducted with the statistical package SAS Version 9.2. Centre based childcare services providing both baseline and follow-up data were included in the analysis of trial outcomes. The median score of the service postcode for the state based on the Socio-Economic Indexes for Areas [60] was used to classify services as being from either high (at or above median) or low (below median) socioeconomic areas. The service postcode was also used to classify the services as either being in a major city, inner regional, outer regional or remote area using the Accessibility/Remoteness Index of Australia [54].

Based on their responses to the survey items, centre based childcare services were classified as implementing fundamental movement skills sessions to a recommended standard if they reported that such programs were implemented daily and always included all of the following components: warm up, cool down, skill specific feedback, extension and challenge experiences, and, modeling and demonstration (based on the NSW Ministry of Health *Munch and Move©* Resource Manual [61]. The formation of other trial outcomes, based on participant responses to survey items is described in Table 1.

Bivariate analyses (Chi Square tests) for categorical variables and paired t-tests for continuous variables were undertaken to determine within group changes in the prevalence of childcare service practices between baseline and follow-up in the intervention and comparison areas.

Multivariate logistic and linear regression models were developed, within a generalized estimating equation framework, to determine between group differences in the change in prevalence of each of the outcome measures from baseline to follow-up. The logistic regression models included terms for time, group (intervention or comparison area) and the interaction of time and region. A p-value of 0.05 for the interaction term was used to determine if there was a statistically significant difference in change in prevalence between the intervention and comparison areas. The characteristics of services were not adjusted for in the logistic regression models as the primary trial objective was to assess change within services and the baseline score of the services effectively controlled for potential differences in baseline characteristics between the two areas.

The sample size for the study was calculated to enable the detection of an absolute difference in the prevalence of policies or practices of 15% between groups with 80% power and an alpha of 0.05. The sample size calculation was based on a conservative assumption of a 50% policy or practice prevalence in the comparison group at follow-up. While the trial sought to assess the policies and practices of all 338 services in the intervention region, a 75% participation and a 25% study attrition rate was estimated based on previous research experience of the authors in this setting, leaving 190 intervention services providing data at follow-up. Based on such study participation and attrition rates, a sample of 268 services from the control group were invited to participate, which was expected to yield the 150 services at follow-up required to detect an effect size of 15% difference in service physical activity policies and practices.

## Results

### Sample

Figure 1 describes study participation and attrition rates. In the intervention region, 275 services completed baseline data collection representing an 81% response rate from eligible services. Of these 228 services (83%) provided follow-up data. In the comparison area, 209 services of all those eligible completed baseline data collection, and of these, 164 (78%) provided follow-up data. Descriptive characteristics of the intervention and comparison services that completed evaluation telephone interviews at both time points and were included in the final analysis are shown in Table 2.

**Table 2:** Baseline characteristics of services included in physical activity outcome analyses by area

**Variable**	**Intervention Area**	**Comparison Area**	P *
Services in high socioeconomic area (%, 95% CI)	41(37, 46)	68 (62, 73)	<0.01
Service geographic locality (%, 95% CI)			
Major city	37 (32,41)	67 (62,63)	<0.01
Inner regional	31 (27,25)	21 (17,26)	<0.01
Outer regional	29 (25,33)	8 (5,11)	<0.01
Remote	3 (1, 4)	2 (0, 3)	<0.01
Services with children of Aboriginal background (%, 95% CI)	71 (66,75)	43 (37,48)	<0.01
Number of children enrolled (mean , 95% CI)	83.6 (78.2, 89.0)	79.9 (72.6, 87.2)	0.42
Hours open (mean , 95% CI)	8.7 (8.5, 9.0)	9.2 (8.9, 9.5)	0.03
Days open (mean , 95% CI)	4.8 (4.7, 4.9)	4.9 (4.8, 5.0)	0.12
Tertiary educated staff (mean , 95% CI)	1.3(1.1, 1.4)	1.0 (1.1, 1.5)	0.83
Contact staff per day (mean , 95% CI)	6.0 (5.7, 6.3)	6.0 (5.6, 6.4)	0.94

*Categorical variables are compared using chi squared tests and continuous variables are compared using t tests.

Services in the intervention area were significantly less likely to be in high socioeconomic areas or located in major cities, had a significantly higher prevalence of services with children of Aboriginal background compared with services in the comparison area (all p = <0.01) and were open for fewer hours per day (p = 0.03). There was a difference, approaching significance, in the mean number of child enrollments (p = 0.06) between services providing baseline data only and those providing both baseline and follow-up data. There were no other differences in the service characteristics of services providing follow-up data and those that did not (p = 0.58-0.95).

### Physical activity promoting practices and service manager knowledge

Table 3 shows the prevalence of practices that promote physical activity in both the intervention and comparison areas. The bivariate within group analyses identified significant pre- post increases for four of the eight outcomes of interest in the intervention area. There were no significant pre-post differences for any outcome in the comparison area.

**Table 3:** Changes in physical activity practices and service manager knowledge of physical activity recommendations over time by area

	**Intervention area**			**Comparison area**			
**Outcome**	**Baseline**	**Follow-up**	**p**^**1**^	**Baseline**	**Follow-up**	**P**^**2**^	**Interaction P**^**3**^
	**2009**	**2010**		**2009**	**2010**		
1. Services with a physical activity policy	21%	49%	<0.01*	34%	38%	0.31	<0.01
a) Physical activity policy referring to child fundamental movement skills development	86%	87%	0.77	80%	85%	0.42	0.72
b) Physical activity policy referring to limits on small screen recreation and TV	45%	82%	<0.01*	60%	65%	0.54	<0.01
c) Physical activity policy referring to physical activity training for staff	63%	86%	<0.01*	60%	68%	0.38	0.07
2. Services conducting daily fundamental movement sessions with recommended components	13%	21%	<0.01*	13%	12%	0.87	0.08
3. Time spent on structured physical activities (mean hours, standard deviation)	1.3 (1.0)	1.5 (1.0)	0.02*	1.5 (1.1)	1.6 (1.0)	0.25	0.65
4. Services where all staff usually participate in free active play (role modeling)	58%	65%	0.09	61%	69%	0.13	0.95
5. Services where all staff usually provide verbal prompts for physical activity	72%	74%	0.52	69%	72%	0.44	0.90
6. Services where children are allowed to watch Small screen recreation less than once per week	23%	22%	0.73	19%	17%	0.62	0.89
7. Services where children participate in seated activities for no longer than 30 minutes at a time	62%	63%	0.84	59%	62%	0.64	0.82
8. Services with staff trained in physical Activity	29%	76%	<0.01*	37%	43%	0.21	<0.01

^1^ Pre-post changes in adoption of physical activity promoting practices for services in the intervention area.

^2^ Pre-post changes in adoption of physical activity promoting practices for services in the comparison area.

^3^ Changes in adoption of physical activity promoting practices between intervention and comparison groups at follow-up (group x time interaction).

Based on the multivariate analyses, adjusting for time and region, relative to the comparison area, intervention area services had significantly greater increases in the proportion with a written physical activity policy (p < 0.01); with policy content referring to placing limits on small screen recreation (p < 0.01); and with staff trained in physical activity (p < 0.01) (Table 3). In addition, the change in proportions between groups trended towards being significantly greater in the intervention compared with the comparison area for two further outcomes: the proportion of services providing fundamental movement skills sessions with the recommended components daily (p = 0.08) and having a policy that refers to physical activity training for staff (p = 0.07). There were no other significant between group differences.

For the intervention area bivariate within group analyses identified a significant pre- post increase in service manager knowledge of the maximum recommended time children should be sedentary (5.4 -11%, p = 0.02) and service manager knowledge of recommendations for participation in physical activity trended towards a significant increase (14 -21%, p = 0.06). For the comparison region, service manager knowledge of physical activity recommendations significantly decreased pre-post for service manager knowledge of maximum recommended time children should watch television (46-32%, p = 0.01) and maximum recommended time children should be sedentary (11–2.5 percent, p < 0.01). Multivariate analyses identified services in the intervention area as having significantly greater increases in service manager knowledge of recommendations for child participation in physical activity relative to the comparison area (p < 0.01). There were no other significant differences in assessment of service manager knowledge between groups.

### Reach and acceptability of the intervention implementation strategies

The majority of service managers in the intervention area (96%) indicated that they would recommend the program to other services (Table 4). Furthermore, 89% of services responded that children in their service were perceived to have benefited from participation in the program. With regard to the acceptability of intervention implementation strategies and resources, 94% of managers indicated that they would recommend the staff training to other services while 49% found the support calls very useful in helping their service to implement the program (Table 4). A total of 68% of managers found the resource kit very useful.

**Table 4:** Reach and acceptability of intervention implementation strategies

**Description**	**Measure**	
Reach	Service received the resource kit	100%
	Services received the newsletters and support emails/faxes.	100%
	Services with staff attending training session	82%
	Services that participated in two support calls	78%
Acceptability	Service manager would recommend the program to other services	94%
	Service manager would recommend training to other services	96%
	Children attending service have benefited from the GFK PA program	89%
	Found the resource kit very useful	68%
	Support calls were very useful in helping our service implement best practice physical activity strategies	91%

% includes services completing baseline and follow up assessments that were included in final analysis.

## Discussion

This is one of only a handful of studies examining the impact of an intervention to increase centre based childcare service’s adoption of policies and practices known to be associated with increased child physical activity. The study extends previous research through its examination of the effectiveness of an intervention delivered setting wide in centre based childcare services in a large and diverse geographic region.

The study found significant within group pre- post increases in the prevalence of four of eight practices in the intervention area and no increases in the comparison area. Significantly greater increases were found in the proportion of services adopting two practices relative to the comparison region: a physical activity policy (including the policy referring to placing limits on small screen recreation) and staff trained in physical activity. In addition, non-significant trends (p =0.07,0.08) towards greater increases in the prevalence of services having a physical activity policy that refers to promoting physical activity training for staff and implementing fundamental movement skills sessions daily in the intervention area were evident.

Similar to the findings previously reported by Hardy and colleagues, the intervention examined in this study was successful in increasing the adoption of some physical activity policies and practices [37]. While the current study employed a broader range of intervention implementation strategies, a number of similarities between intervention components of the two studies were evident such as the inclusion of staff training, program resources and instructional materials, two follow-up support contacts and incentives. However, the study by Hardy and colleagues was conducted as an efficacy trial, in a selected and small sample of government preschools only, not long day care centers. The current study was conducted as a component of a program delivered to all services of both types, and sought to determine the effectiveness of the intervention as a program dissemination/adoption strategy [62]. The finding of a significant increase in the adoption of a number of childcare service practices in such circumstances suggests that the intervention approach has the potential to be utilized more broadly as a means of translating research evidence into practice [63].

As the intervention was not effective in producing increases in the prevalence of all targeted practices, additional strategies that are intensive or more prolonged, or some combination of these may be needed to achieve more comprehensive changes to the physical activity promoting practices of services. In addition, several factors may have limited the effectiveness of the practice change intervention and could be considered as opportunities for enhancing the implementation of such an intervention in the future. First, the intervention did not involve all staff within each service receiving training. Workshop attendance was limited to two staff from each childcare service, and few additional staff were found to have utilized the on-line training module despite project records indicating that 80% had access to the internet at the service. In addition, 22% of services did not participate in both follow-up calls, predominately as service managers could not be contacted by intervention staff within ten call attempts or service managers chose not receive the telephone support. Furthermore, the percentage of service managers with correct knowledge of sedentary and physical activity recommendations was relatively low, both at baseline and follow-up (5.4-21%).

These findings suggest that such intervention components may not have overcome frequently cited barriers such a staff time constraints which are known impediments to service staff engagement in health promoting practices [64]. Supportive attitudes, knowledge and skills of all staff are important determinants of organizational improvement and likely to be fundamental to the success of practice change initiatives [65]. Providing training to all staff in a service by incorporating training as part of a mandatory component of staff induction, the inclusion of refresher training in annual staff development opportunities and increased emphasis on knowledge and attitudes as well as skills may represent an opportunity for improving the long term impact of such implementation initiatives without placing additional time demands on staff [66].

Second, the intervention involved two follow-up telephone support contacts over a three month period after the initial training. Research from other settings including schools suggests that practice change requires support over a period of three to four years [67,68]. In addition, early childhood educational research suggests that prolonged periods of ongoing support (at least 12 months), is required for the embedding of new and complex teaching practice change in this setting [69]. Providing ongoing support through on-site visits [65] and/or the establishment of supportive networks to provide peer support for practice changes, may represent a sustainable, low cost option of providing prolonged practice change support [38,69,70]. Third, the effectiveness of the intervention could have been enhanced through the inclusion of additional intervention components found to be effective in practice change initiatives implemented in other settings. For example, embedding service delivery practices or practice change elements in organizational procedures and systems that prompt and monitor their delivery [70,71] or including them in regulatory standards of care has been shown to be effective, particularly in health service quality improvement initiatives [72]. As such, integrating physical activity within routine daily staff activity programming [73], and including the promotion of child physical activity in licensing and accreditation processes for services may also facilitate greater adoption of physical activity promoting characteristics in this setting.

Finally, opportunities for enhancing the quality and perceived relevance of intervention support and resources provided to services may result through greater tailoring of such support [70]. This may include greater targeting of strategies for rural or remote services, services in disadvantaged areas or with high aboriginal child enrollments; targeting strategies based on service readiness to change and identifying and providing support to address other individual staff and organizational impediments to policy or practice adoption [74]. The need for such a focus is suggested by findings in this study that half of the services perceived the follow up support call to be only somewhat or not at all useful.

A strength of this study was its high external validity due to the broad inclusion criteria, and high participation and retention rates. A number of limitations of the study, however, warrant consideration. The primary limitation of the trial was its reliance on the self report of service managers for the measurement of the prevalence of service policies and practices. Direct observation, recommended as the gold standard for environmental assessments [33], was considered prohibitively expensive and impractical given the scale of the intervention. While the validity of service manager reports in this study are unknown, previous research indicates that childcare managers and school principals can accurately report the health promotion practices of their organizations [58,75]. A further limitation of the study was the concurrent roll-out of a government sponsored program in the comparison area (*Munch and Move©*) during the study period. Twenty three percent of service managers in the comparison area reported that they had any staff attend *Munch and Move©* training at follow-up. The estimated intervention effect size reported in this study may have been larger had comparison services not received such support. Also the study examined only physical activity promoting policies and practices targeting children 3–5 years. Future research may consider evaluating the impact of an intervention on the adoption of practices supporting activity of infants and younger children. Finally, the study did not employ a randomized evaluation design. For this study, which was conducted in the context of whole of population child obesity prevention program, random assignment was not feasible. Nonetheless, the use of randomized experimental deigns may improve the internal validity of future trials.

The findings of this trial provide an important contribution to the limited literature regarding the implementation of population-wide government funded obesity-prevention programs generally, and the adoption of such programs in childcare services setting in particular. The findings showed the intervention was effective in improving a number of centre based childcare service policies and practices associated with promoting child physical activity. Adoption of a broader range of practices may require more intensive and prolonged intervention support.

## Competing interests

The authors declare that they have no competing interests.

## Author’s contributions

First author MFinch led the development of this manuscript. Authors LW, MFinch, DE, MFalkiner and NP conceived the intervention. Authors LW, JW, LH and AJM contributed to the research design and trial methodology. All authors contributed to, read and approved the final version of this manuscript.

## References

[B1] Janz KF Letuchy EM Eichenberger Gilmore JM Burns TL Torner JC Willing MC Levy SM Early Physical Activity Provides Sustained Bone Health Benefits Later in Childhood Med Sci Sports & Exerc 2010 42 6 1072 1078 19997029 10.1249/MSS.0b013e3181c619b2 PMC2874089

[B2] Cliff DP Okely AD Smith LM Kim M Relationships Between Fundamental Movement Skills and Objectively Measured Physical Activity in Preschool Children Pediatr Exerc Sci 2009 21 4 436 449 20128363 10.1123/pes.21.4.436

[B3] Trost SG Sirard JR Dowda M Pfeiffer KA Pate RR Physical activity in overweight and no overweight preschool children Int J Obes Relat Metab Disord 2003 27 7 834 839 10.1038/sj.ijo.0802311 12821970

[B4] Timmons BW Naylor P-J Pfeiffer KA Physical activity for preschool children–how much and how? Canadian Journal of Public Health Revue Canadienne de Sante Publique 2007 98 Suppl 2 S122 S134 18213943

[B5] Vandewater E Rideout V Wartella E Huang X Lee J Shim M Digital childhood: Electronic media and technology use amog infants, toddlers, and preschoolers Pediatrics 2007 119 e1006 e1015 10.1542/peds.2006-1804 17473074

[B6] Tucker P The physical activity levels of preschool-aged children: A systematic review Early Childhood Research Quarterly 2008 23 4 547 558 10.1016/j.ecresq.2008.08.005

[B7] Vale S Silva P Santos R Soares-Miranda L Mota J Vale S Silva P Santos R Soares-Miranda L Mota J Compliance with physical activity guidelines in preschool children J Sports Sci 2010 28 6 603 608 10.1080/02640411003702694 20397094

[B8] Hinkley T Salmon J Okely AD Crawford D Hesketh K Preschoolers' Physical Activity, Screen Time and Compliance with Recommendations Med Sci Sports & Exerc 2012 44 3 458 465 10.1249/MSS.0b013e318233763b 21900847

[B9] Australian Bureau of Statistics Childhood Education and Care June 2008 (Reissue) 2009 Canberra: Australian Bureau of Statistics Cat no.: 4402.0

[B10] Benjamin SE Haines J Ball SC Ward DS Improving Nutrition and Physical Activity in Child Care: What Parents Recommend J Am Diet Assoc 2008 108 11 1907 1911 10.1016/j.jada.2008.08.018 18954582

[B11] Ward DS Physical activity in young children: the role of child care Med Sci Sports Exerc 2010 42 3 499 501 10.1249/MSS.0b013e3181ce9f85 20068500

[B12] Story M Kaphingst KM French S The role of child care settings in obesity prevention Future Child 2006 16 1 143 168 10.1353/foc.2006.0010 16532662

[B13] Lawlis T Mikhailovich K Morrison P Healthy eating and physical activity programs, resources and staff training in long day care and family day care settings: A Literature Review 2006 Canberra: Healthpact Research Centre for Health Promotion and Wellbeing

[B14] Dowda M Brown WH McIver KL Pfeiffer KA O'Neill JR Addy CL Pate RR Policies and characteristics of the preschool environment and physical activity of young children Pediatrics 2009 123 2 e261 266 10.1542/peds.2008-2498 19171578 PMC2632768

[B15] NSW Department of Family and Community Services Children (Education and Care Services) Supplementary Provisions Regulation 2004 Ashfield: NSW Government http://www.legislation.nsw.gov.au/fullhtml/inforce/subordleg+260+2004+FIRST+0+N

[B16] National Childcare Accreditation Council Quality Improvement and Accreditation System. Quality Trends Report 2010 http://www.ncac.gov.au/reports_statistics/past_reports.asp#qtr

[B17] Dowda M Russell RP Stewart GT Almeida MJCA John RS Influences of preschool policies and practices on childrens physical activity J Community Health 2004 29 3 183 15141894 10.1023/b:johe.0000022025.77294.af

[B18] Boldemann C Blennow M Dal H Mårtensson F Raustorp A Yuen K Wester U Impact of preschool environment upon children's physical activity and sun exposure Prev Med 2006 42 4 301 308 10.1016/j.ypmed.2005.12.006 16448688

[B19] Bower JK Hales DP Tate DF Rubin DA Benjamin SE Ward DS The Childcare Environment and Children's Physical Activity Am J Prev Med 2008 34 1 23 29 10.1016/j.amepre.2007.09.022 18083447

[B20] Cardon GM Cauwenberghe E Labarque V Haerens L De Bourdeaudhuij IM The contribution of playground factors in explaining children's physical activity during recess Int J Behav Nutr Phys Act 2008 5 11 10.1186/1479-5868-5-11 PMC2266778 18302741

[B21] Finn K Johannsen N Specker B Factors associated with physical activity in preschool children J Pediatr 2002 140 1 81 85 10.1067/mpd.2002.120693 11815768

[B22] Trost SG Ward DS Senso M Trost SG Ward DS Senso M Effects of child care policy and environment on physical activity Med Sci Sports Exerc 2010 42 3 520 525 10.1249/MSS.0b013e3181cea3ef 20068496

[B23] Ward DS Vaughn A McWilliams C Hales D Interventions for increasing physical activity at child care Med Sci Sports Exerc 2010 42 3 526 534 10.1249/MSS.0b013e3181cea406 20068495

[B24] Williams H Pfeiffer K O'Neill J Dowda M McIver K Brown W Pate R Motor skill performance and physical activity in preschool children Obesity 2008 16 1421 1426 10.1038/oby.2008.214 18388895

[B25] Okely AD Salmon J Trost SG Hinkley T Discussion paper for the development of physical activity recommendations for children under five years 2008 Canberra: Australian Department of Health and Ageing

[B26] Cashmore A Jones S Growing Up Active: A Study Into Physical Activity in Long Day Care Centers J Res Child Educ 2008 23 2 179 10.1080/02568540809594654

[B27] Gubbels JS Kremers SP van Kann DH Stafleu A Candel MJ Dagnelie PC Thijs C de Vries NK Interaction Between Physical Environment, Social Environment, and Child Characteristics in Determining Physical Activity at Child Care Health Psychol 2011 30 1 84 90 21133542 10.1037/a0021586

[B28] Australian Government Department of Health and Ageing Get Up and Grow: Healthy Eating and Physical Activity for Early Childhood 2009 Canberra: Director/Coordinator Book

[B29] Wolfenden L Neve M Farrel L Lecanthelinais C Bell C Milat A Wiggers J Sutherland R Physical activity policies and practices of childcare centers in Australia J Paediatr Child Health 2010 47 3 73 6 20500433 10.1111/j.1440-1754.2010.01738.x

[B30] McWilliams C Ball SC Benjamin SE Hales D Vaughn A Ward DS Best-Practice Guidelines for Physical Activity at Child Care Pediatrics 2009 124 6 1650 1659 10.1542/peds.2009-0952 19917582

[B31] Copeland KA Sherman SN Khoury JC Foster KE Saelens BE Kalkwarf HJ Wide Variability in Physical Activity Environments and Weather-Related Outdoor Play Policies in Child Care Centers Within a Single County of Ohio Arch Pediatr Adolesc Med 2011 165 5 435 442 10.1001/archpediatrics.2010.267 21199969 PMC3086945

[B32] Larson N Ward DS Neelon SB Story M What Role Can Child-Care Settings Play in Obesity Prevention? A Review of the Evidence and Call for Research Efforts J Am Diet Assoc 2011 111 9 1343 1362 10.1016/j.jada.2011.06.007 21872698

[B33] Ward DS Benjamin SE Ammerman AS Ball SC Neelon BH Bangdiwala SI Nutrition and Physical Activity in Child Care: Results from an Environmental Intervention Am J Prev Med 2008 35 4 352 356 10.1016/j.amepre.2008.06.030 18701236

[B34] Williams CL Bollella MC Strobino BA Spark A Nicklas TA Tolosi LB Pittman BP “Healthy-Start”: Outcome of an Intervention to Promote a Heart Healthy Diet in Preschool Children J Am Coll Nutr 2002 21 1 62 71 11838889 10.1080/07315724.2002.10719195

[B35] Endres J Barter S Theodora P Welch P Soy-enhanced lunch acceptance by preschoolers J Am Diet Assoc 2003 103 346 351 12616257 10.1053/jada.2003.50046

[B36] Ammerman AS Ward DS Benjamin SE Ball SC Sommers JK Molloy M Dodds JM Ammerman AS Ward DS Benjamin SE An intervention to promote healthy weight: Nutrition and Physical Activity Self-Assessment for Child Care (NAP SACC) theory and design Prev Chronic Dis 2007 4 3 A67 17572971 PMC1955393

[B37] Hardy L King L Kelly B Farrell L Howlett S Munch and Move: evaluation of a preschool healthy eating and movement skill program Int J Behav Nutr Phys Act 2010 7 80 10.1186/1479-5868-7-80 21047434 PMC2988057

[B38] Owen N Glanz K Sallis JF Kelder SH Evidence-Based Approaches to Dissemination and Diffusion of Physical Activity Interventions Am J Prev Med 2006 31 4S S35 S44 16979468 10.1016/j.amepre.2006.06.008

[B39] NSW Department of Health HealtheResource_Demography 2010 http://www2.hnehealth.nsw.gov.au/hneph/HHNE/dem/demIntro.htm

[B40] Australian Bureau of Statistics 2006 Census of Population Health and Housing 2007 Canberra: Australian Bureau of Statistics

[B41] Australian Bureau of Statistics National Regional Profile: New South Wales http://www.abs.gov.au/AUSSTATS/abs@nrp.nsf/Latestproducts/1Population/People12006-2010?opendocument&tabname=Summary&prodno=1&issue=2006-2010&num=&view

[B42] Australian Government of Department and Education National Quality Framework for Early Childhood Education and Care http://www.deewr.gov.au/Earlychildhood/Policy_Agenda/Quality/Pages/home.aspx

[B43] Trost SD Interventions to Promote Physical Activity in Young Children Encyclopedia on Early Child Dev online 2011 1 6

[B44] Fees B Trost S Bopp M Dzewaltowski DA Physical Activity Programming in Family Child Care Homes: Providers' Perceptions of Practices and Barriers J Nutr Educ Behav 2009 41 4 268 273 10.1016/j.jneb.2008.01.013 19508932

[B45] McCullum C Hoelscher DM Eagen JM Kelder SH Barroso CS Evaluation of the Dissemination and Implementation of the CATCH Eat Smart School Nutrition Program in Texas J Child Nutr Manag 2004 1 spring http://docs.schoolnutrition.org/newsroom/jcnm/04spring/mccullum/

[B46] Schofield M Edwards K Pearce R Effectiveness of two strategies for dissemination of sun-protection policy in New South Wales primary and secondary schools Aust N Z J Public Health 1997 21 7 743 750 10.1111/j.1467-842X.1997.tb01791.x 9489193

[B47] Abraham C Michie S Abraham C Michie S A taxonomy of behavior change techniques used in interventions Health Psychol 2008 27 3 379 387 18624603 10.1037/0278-6133.27.3.379

[B48] Soumerai SB Avorn J Principles of educational outreach ('academic detailing') to improve clinical decision making JAMA 1990 263 4 549 556 10.1001/jama.1990.03440040088034 2104640

[B49] Jamtvedt G Young JM Kristoffersen DT O’Brien MA Oxman AD Audit and feedback: effects on professional practice and healthcare outcomes Cochrane Database of Systematic Reviews 2012 6 Art. No.: CD000259 10.1002/14651858.CD000259.pub3 PMC11338587 22696318

[B50] Grol R Wensing M What drives change? Barriers to and incentives for achieving evidence-based practice Med J Aust 2004 180 Supplement 6 57 60 15012583 10.5694/j.1326-5377.2004.tb05948.x

[B51] Stone EG Morton SC Hulscher ME Maglione MA Roth EA Grimshaw JM Mittman BS Rubenstein LV Rubenstein LZ Shekelle PG Interventions that increase use of adult immunization and cancer screening services: a meta-analysis. [Summary for patients in Ann Intern Med. 2002 May 7;136(9):I16; PMID: 11992319] Ann Intern Med 2002 136 9 641 651 11992299 10.7326/0003-4819-136-9-200205070-00006

[B52] Hardy L Farrell L King L Howlett S Cumming G Munch and Move Implementation and Evaluation, Phase 1 (2008–2009) report 2009 Sydney: Prevention Research Collaboration

[B53] NSW Department of Health About Munch and Movehttp://www.healthykids.nsw.gov.au/campaigns-programs/about-munch-move.aspx

[B54] Department of Health and Aged Care Measuring remoteness. Accessibility/Remoteness Index of Australia (ARIA) 2001 Department of Health and Aged Care (Rev. 2001) Occasional papers, Canberra: Information and Research Branch

[B55] Australian Bureau of Statistics An introduction to SocioEconomic Indexes for Areas (SEIFA) 2006 Canberra: Australian Bureau of Statistics Cat. no.: 2039.0

[B56] Australian Institute of Health and Welfare Rural, regional and remote health: A guide to remoteness classifications 2004 Canberra: AIHW vol. AIHW cat. no. PHE 53

[B57] Ammerman AS Benjamin SE Sommers JS Ward DS The Nutrition and Physical Activity Self-Assessment for Child Care (NAP SACC) environmental self-assessment instrument 2004 Chapel Hill, NC: NC DHHS and the Center for Health Promotion and Disease Prevention

[B58] Benjamin SE Neelon B Ball SC Bangdiwala SI Ammerman AS Ward DS Reliability and validity of a nutrition and physical activity environmental self-assessment for child care Int J Behav Nutr Phys Act 2007 4 29 10.1186/1479-5868-4-29 17615078 PMC1934916

[B59] Australian Government Department of Health and Aged Care National Physical Activity Recommendations for Children 0–5 years olds 2010 http://www.health.gov.au/internet/main/publishing.nsf/Content/phd-physical-activity-0-5-pdf-cnt.htm

[B60] Australian Bureau of Statistics Census of Population and Housing: Socio-Economic Indexes for Areas (SEIFA), Australia - Data only 2006–2008 Canberra: Australian Bureau of Statistics Cat no.: 2033 0 55 001

[B61] NSW Ministry of Health Munch and Move resource manual: Birth to five years 2012 North Sydney

[B62] Nutbeam D Bauman A Evaluation in a Nutshell 2006 Sydney: McGraw Hill

[B63] Reynolds KD Spruijt-Metz D Translational research in childhood obesity Eval Health Prof 2006 29 219 10.1177/0163278706287346 16645185

[B64] Petrunoff N Lloyd B Watson N Morrisey D Suitability of a structured Fundamental Movement Skills program for long day care centers: a process evaluation Health Promot J Austr 2009 20 1 65 68 19402818 10.1071/he09065

[B65] Rohrbach LA Grana R Sussman S Valente TW Rohrbach LA Grana R Sussman S Valente TW Type II translation: transporting prevention interventions from research to real-world settings Eval Health Prof 2006 29 3 302 333 10.1177/0163278706290408 16868340

[B66] Fukkink RG Lont A Does training matter? A meta-analysis and review of caregiver training studies Early Child Res Q 2007 22 294 311 10.1016/j.ecresq.2007.04.005

[B67] International Union for Health Promotion and Education Achieving health promoting schools: guidelines for promoting health in schools 2006 Saint-Denis Cedex http://www.iuhpe.org/uploaded/Publications/Books_Reports/HPS_GuidelinesII_2009_English.pdf

[B68] Oxman AD Thomson MA Davis DA Haynes RB No magic bullets: a systematic review of 102 trials of interventions to improve professional practice Can Med Assoc J 1995 153 10 1423 1431 7585368 PMC1487455

[B69] Moulding NT Silagy CA Weller DP A framework for effective management of change in clinical practice: dissemination and implementation of clinical practice guidelines Qual Health Care 1999 8 3 177 183 10.1136/qshc.8.3.177 10847875 PMC2483658

[B70] Mitchell L Cubey P Characteristics of professional development linked to enhanced pedagogy and children’s learning in early childhood settings:Best Evidence Synthesis 2003 Wellington: New Zealand Council for Educational Research

[B71] Grimshaw JM Shirran L Thomas R Mowatt G Fraser C Bero L Grilli R Harvey E Oxman A O'Brien MA Changing provider behavior: an overview of systematic reviews of interventions Med Care 2001 39 8 Suppl 2 II2 45 11583120

[B72] Cockburn J Adoption of evidence into practice: can change be sustainable? Med J Aust 2004 180 Supplement 6 66 67 10.5694/j.1326-5377.2004.tb05951.x 15012586

[B73] Edvardsson K Garvare R Ivarsson A Eurenius E Mogren I Nystrom ME Sustainable practice change: professionals' experiences with a multisectoral child health promotion programme in Sweden BMC Health Serv Res 2011 11 61 10.1186/1472-6963-11-61 21426583 PMC3077331

[B74] Grol R Grimshaw J From best evidence to best practice: effective implementation of change in patients’ care Lancet 2003 362 1225 1230 10.1016/S0140-6736(03)14546-1 14568747

[B75] Schofield MJ Solar protection issues for schools: policy, practice and recommendations Aust J Public Health 1991 15 135 141 1912056 10.1111/j.1753-6405.1991.tb00323.x

